# Engagement and task satisfaction during cognitive stimulation tasks facilitated by a social robot versus a human research assistant

**DOI:** 10.1002/alz70858_103934

**Published:** 2025-12-26

**Authors:** Monica Sanches Yassuda, Raquel Ribeiro de Oliveira, Patrícia Bet, Maria Paulina Berrido da Silva, Isabella de Brito Ungaro, Sarajane Marques Peres, Ruth Caldeira de Melo, Marcelo Fantinato, Meire Cachioni

**Affiliations:** ^1^ University of São Paulo, São Paulo, São Paulo, Brazil

## Abstract

**Background:**

Social robots have emerged as cutting‐edge technology in the care of older adults, with potential applications in cognitive stimulation. However, the interactive capabilities of these robots remain limited. This study aimed to compare engagement and satisfaction among long‐term care home (LTCH) residents during cognitive activities facilitated by a social robot versus a human research assistant.

**Method:**

The study utilized the social robot Robios, programmed in Java. A total of 24 residents (13 women; mean age = 80.4 years, SD = 9.3; mean education = 8.8 years, SD = 4.4) with cognitive impairment or dementia (mean MMSE = 20.2, SD = 4.8) participated. Each resident completed two applications (Memory Game and Music Quiz) with Robios and with a human research assistant in a counterbalanced design. Engagement with tasks in both modalities was assessed using a semi‐structured questionnaire based on the Menorah Park Engagement Scale (0‐10 points), derived from the observations of two independent research assistants. Additionally, participants rated their satisfaction with the activities (0‐5 points) after completing them with Robios and the human research assistant. The Wilcoxon signed‐rank test was used to compare engagement and satisfaction across the two modalities (Robios vs. Human).

**Result:**

Engagement was significantly higher during the Memory Game when facilitated by Robios (mean = 9.2, SD = 1.0 vs. mean = 8.3, SD = 0.9, *p* = 0.009). Similarly, for the Music Quiz, engagement was greater with Robios (mean = 9.2, SD = 0.8 vs. mean = 8.7, SD = 0.6, *p* = 0.02). However, no significant differences were observed in task satisfaction between the two modalities for either the Memory Game or the Music Quiz.

**Conclusion:**

Social robots seem to enhance engagement among LTCH residents during cognitive stimulation tasks. However, task satisfaction remains comparable when these activities are completed with either a social robot or a human research assistant.

